# Husband 

**DOI:** 10.3201/eid1407.080568

**Published:** 2008-07

**Authors:** Connie Smith Watson

She always brushed her hair back
Sternly, twisting one small knot about the crown.
He did not like the plain, broad brow.
“I hate that tightness,” he would mutter to himself.
But sometimes he would stoop to kiss her forehead
And at midnight, hesitatingly, her lips,
Surprised each time the way her arms would reach for him.
When little Thomas had diphtheria at two,
Her back was hourly bent above the crib,
And one day as he stood beside her
He too bent, drawn helplessly, and
Kissed the tender whiteness of her neck,
The unruly tendrils capturing his lips.
With frost-cracked palm he stroked
Her disciplined small head,
And never thought to mutter any more.

